# Supplementary material for: Bidirectional regulation of the brain-gut axis in Macaca mulatta: implications for wildlife conservation and experimentation
Source: Microbiol Spectr. 2025 Jul 28;13(9):e01338-25. doi: 10.1128/spectrum.01338-25 (PMC12403777; doi:10.1128/spectrum.01338-25)
Supplement: Supplemental material — Fig. S1 and S2; Tables S1 and S2. [file spectrum.01338-25-s0001.docx]

**Supplemental Material**


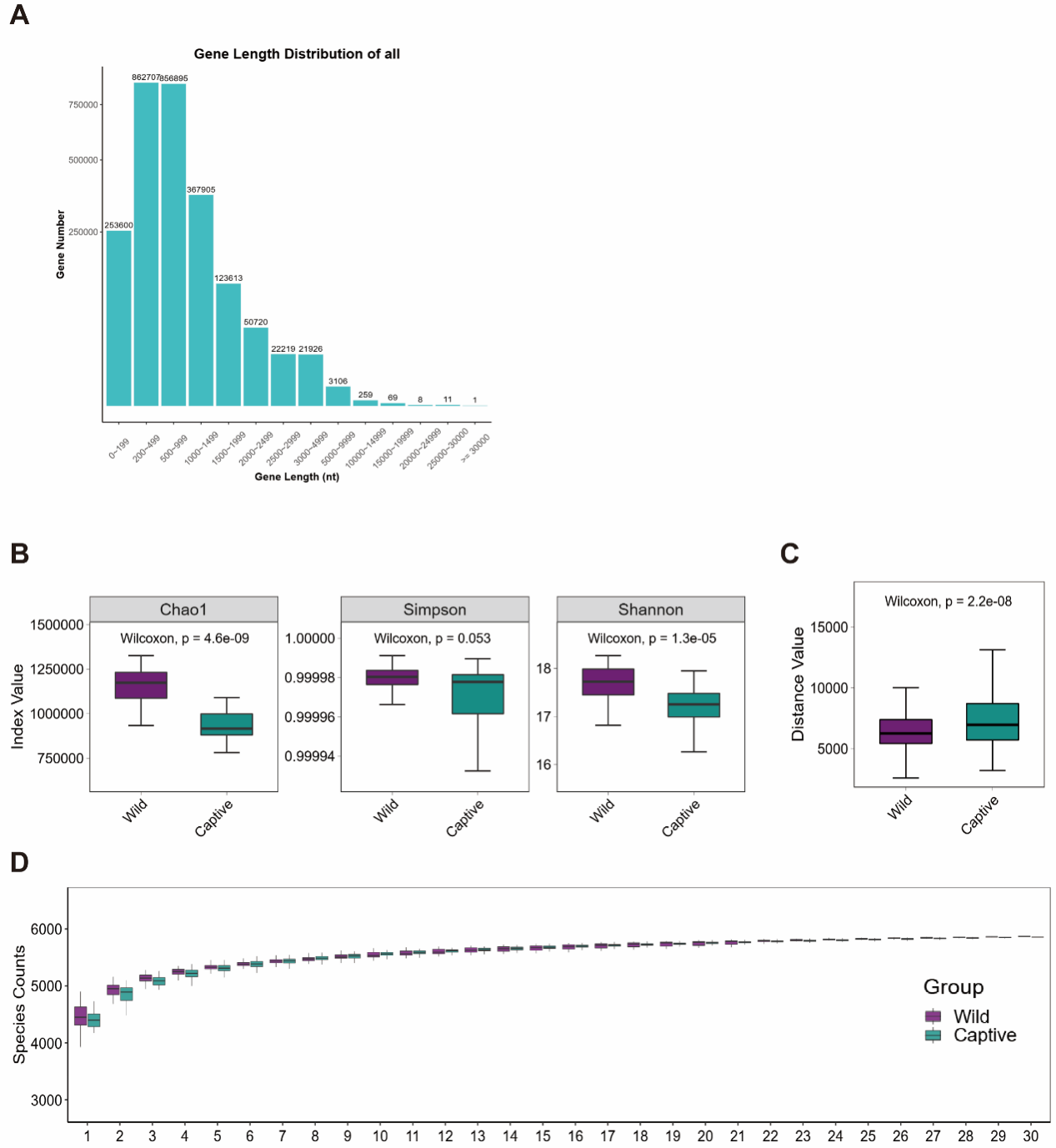


**Supplementary Figure 1. Analysis of Gut Microbiota Gene Diversity and Species Differences**

A. Distribution of gene fragment lengths. The x-axis represents the length intervals of genes, and the y-axis represents the number of genes falling within each length interval.

B. Gene Alpha diversity boxplot. Each boxplot represents a diversity index, with the x-axis indicating the group and the y-axis representing the index value.

C. Gene Beta diversity boxplot. The x-axis and the color of the boxes represent different groups, and the y-axis indicates the distance between samples.

D. Species rarefaction curve boxplot. The x-axis represents the number of samples, and the y-axis represents the number of species (number of detected species), with the color of the boxes representing the group.


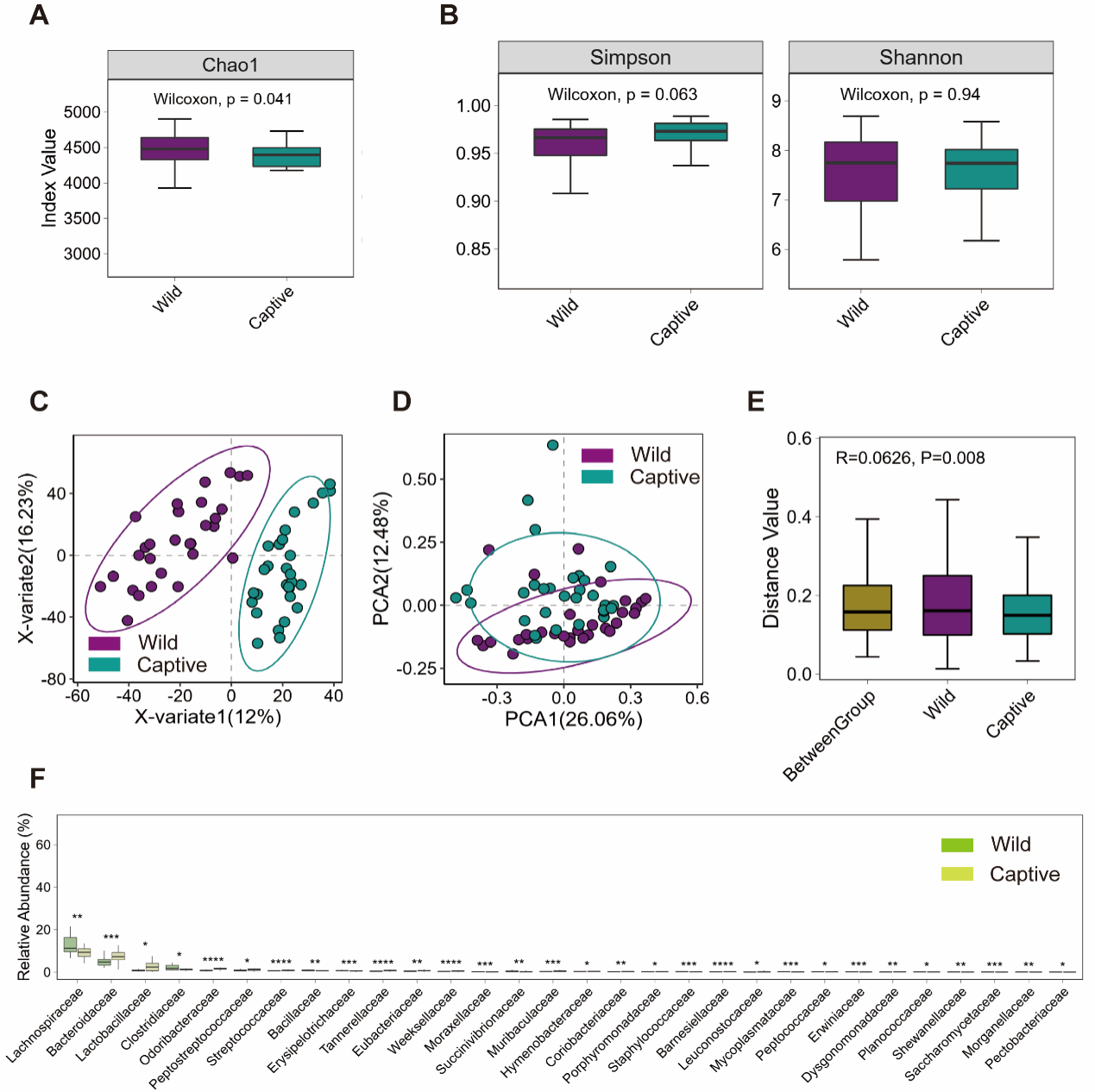


**Supplementary Figure 2. Differential Analysis and Annotation of Microbial Metabolites in Macaques**

A. Alpha diversity Chao1 boxplot for species. The x-axis represents the group, and the y-axis represents the index value. p < 0.05 indicates significant differences in the Alpha index between groups.

B. Alpha diversity boxplot for species. The x-axis represents the group, and the y-axis represents the index value.

C. PLS-DA scatter plot. Each point represents a sample, with blue points indicating the Captive group and yellow points indicating the Wild group. The x- and y-axes represent the PLS dimensions that separate the samples, and the values in the axis titles (in parentheses) indicate the proportion of sample variance explained by the reduced dimensions.

D. PCA clustering plot. Each point represents a sample, with different colors indicating different groups.

E. Anosim analysis plot for species. The y-axis represents the distance value, with "BetweenGroup" indicating the distance between groups. R is the Anosim statistic, and p < 0.05 indicates significant differences between groups.

F. Boxplot of species differences at the species level.

**Supplementary Table 1. Metagenomic Sequencing Quality Control and Post-Host Removal Sequencing Results**

| **Sample ID** | **Raw Reads** | **Clean Reads** | **Clean data rate**  **（%）** | **Q20**  **(%)** | **Q30**  **(%)** | **GC Content (%)** | **Reads After Host Removal** |
| --- | --- | --- | --- | --- | --- | --- | --- |
| LS1 | 45,760,000 | 40,145,022 | 87.73 | 97.25 | 93.71 | 46.27 | 40,038,430 |
| LS2 | 43,200,000 | 40,116,222 | 92.86 | 97.05 | 93.01 | 44.56 | 35,045,186 |
| LS3 | 42,880,000 | 40,245,630 | 93.86 | 97.37 | 93.82 | 45.34 | 40,202,256 |
| LS4 | 44,480,000 | 40,133,010 | 90.23 | 97.25 | 93.24 | 45.09 | 40,016,668 |
| LS5 | 41,680,000 | 40,090,280 | 96.19 | 96.72 | 91.94 | 44.97 | 39,995,426 |
| LS6 | 42,000,000 | 40,096,088 | 95.47 | 97.07 | 93.45 | 45.88 | 39,594,668 |
| LS7 | 42,240,000 | 40,174,822 | 95.11 | 96.79 | 93.31 | 49.36 | 40,121,208 |
| LS8 | 41,920,000 | 40,187,990 | 95.87 | 97.03 | 93.35 | 45.78 | 39,788,378 |
| LS9 | 42,560,000 | 40,130,038 | 94.29 | 96.95 | 92.85 | 45.85 | 40,013,948 |
| LS10 | 42,560,000 | 40,272,342 | 94.62 | 96.98 | 92.87 | 46.01 | 40,106,840 |
| LS11 | 43,200,000 | 40,262,106 | 93.2 | 97.09 | 93.4 | 47.9 | 40,182,312 |
| LS12 | 42,880,000 | 40,187,228 | 93.72 | 97.19 | 93.62 | 46.19 | 39,695,680 |
| LS13 | 41,920,000 | 40,259,234 | 96.04 | 96.89 | 92.68 | 45.72 | 40,241,022 |
| LS14 | 44,160,000 | 40,070,744 | 90.74 | 96.97 | 93.08 | 46.63 | 39,885,158 |
| LS15 | 43,200,000 | 40,150,952 | 92.94 | 96.91 | 92.99 | 46.67 | 37,662,742 |
| LS16 | 42,960,000 | 40,141,208 | 93.44 | 98.14 | 94.7 | 47.12 | 40,118,480 |
| LS17 | 44,560,000 | 40,190,282 | 90.19 | 98.28 | 94.63 | 45.05 | 40,167,548 |
| LS18 | 41,760,000 | 40,199,026 | 96.26 | 97.97 | 94.3 | 46.91 | 39,427,056 |
| LS19 | 43,680,000 | 40,069,918 | 91.74 | 98.22 | 94.52 | 45.28 | 39,946,986 |
| LS20 | 42,080,000 | 40,148,350 | 95.41 | 98.16 | 94.92 | 48.9 | 39,804,066 |
| LS21 | 43,360,000 | 40,141,468 | 92.58 | 97.72 | 93.3 | 44.97 | 40,121,558 |
| LS22 | 42,640,000 | 40,136,018 | 94.13 | 98.21 | 94.6 | 48.05 | 40,035,898 |
| LS23 | 42,640,000 | 40,185,416 | 94.24 | 97.92 | 94 | 47.23 | 39,983,358 |
| LS24 | 47,920,000 | 40,072,668 | 83.62 | 98.06 | 94.24 | 44.88 | 39,934,702 |
| LS25 | 43,040,000 | 40,236,464 | 93.49 | 97.94 | 93.97 | 46.45 | 40,046,762 |
| LS26 | 44,640,000 | 40,164,534 | 89.97 | 98.16 | 94.7 | 47.31 | 38,869,762 |
| LS27 | 45,840,000 | 40,142,674 | 87.57 | 98.34 | 94.85 | 43.72 | 39,688,148 |
| LS28 | 43,360,000 | 40,102,726 | 92.49 | 98.39 | 95.24 | 46.73 | 40,006,026 |
| LS29 | 42,080,000 | 40,141,748 | 95.39 | 98.03 | 94.56 | 47.45 | 39,990,436 |
| LS30 | 42,000,000 | 40,202,444 | 95.72 | 97.94 | 94.17 | 47.81 | 40,040,386 |

**Continued Supplementary Table 1**

| **Sample ID** | **Raw Reads** | **Clean Reads** | **Clean data rate**  **（%）** | **Q20**  **(%)** | **Q30**  **(%)** | **GC Content (%)** | **Reads After Host Removal** |
| --- | --- | --- | --- | --- | --- | --- | --- |
| BT1 | 43,840,000 | 40,158,340 | 91.6 | 98.3 | 94.84 | 48.96 | 40,129,666 |
| BT2 | 44,400,000 | 40,170,292 | 90.47 | 98.38 | 95.16 | 48.3 | 40,134,540 |
| BT3 | 42,720,000 | 40,187,672 | 94.07 | 98.07 | 94.54 | 49.03 | 40,038,730 |
| BT4 | 43,600,000 | 40,167,578 | 92.13 | 98.17 | 94.76 | 49.73 | 39,646,450 |
| BT5 | 43,280,000 | 40,148,664 | 92.76 | 98.39 | 94.8 | 49.15 | 39,997,700 |
| BT6 | 43,600,000 | 40,143,944 | 92.07 | 98.17 | 94.27 | 48.21 | 38,817,434 |
| BT7 | 42,880,000 | 40,105,986 | 93.53 | 97.86 | 94.43 | 49.12 | 39,676,866 |
| BT8 | 45,280,000 | 40,152,196 | 88.68 | 98.42 | 95.12 | 47.41 | 39,717,522 |
| BT9 | 42,640,000 | 40,080,938 | 94 | 98.2 | 94.09 | 45.49 | 40,041,334 |
| BT10 | 52,400,000 | 40,149,802 | 76.62 | 98.53 | 95.59 | 47.29 | 40,124,056 |
| BT11 | 61,840,000 | 40,082,260 | 64.82 | 98.44 | 95.43 | 46.21 | 40,078,026 |
| BT12 | 46,400,000 | 40,117,888 | 86.46 | 98.48 | 95.3 | 46.56 | 40,083,966 |
| BT13 | 45,280,000 | 40,185,050 | 88.75 | 98.09 | 94.96 | 49.76 | 40,133,810 |
| BT14 | 44,320,000 | 40,178,214 | 90.65 | 98.16 | 94.57 | 47.38 | 40,123,268 |
| BT15 | 42,240,000 | 40,202,308 | 95.18 | 97.92 | 94.27 | 48.95 | 39,860,202 |
| BT16 | 43,520,000 | 40,142,126 | 92.24 | 96.84 | 91.46 | 48.53 | 40,081,694 |
| BT17 | 48,000,000 | 40,026,312 | 83.39 | 97.45 | 92.59 | 45.74 | 40,023,560 |
| BT18 | 42,800,000 | 39,848,462 | 93.1 | 96.59 | 90.98 | 47.45 | 39,831,942 |
| BT19 | 45,120,000 | 40,174,450 | 89.04 | 97.01 | 92.15 | 48.51 | 40,126,390 |
| BT20 | 43,600,000 | 40,079,354 | 91.93 | 97 | 90.61 | 45.98 | 40,054,794 |
| BT21 | 45,440,000 | 40,246,846 | 88.57 | 96.93 | 91.66 | 45.09 | 40,231,976 |
| BT22 | 51,200,000 | 40,208,976 | 78.53 | 97.19 | 92.72 | 47.83 | 40,178,906 |
| BT23 | 46,080,000 | 40,150,888 | 87.13 | 97.2 | 92.41 | 45.16 | 40,127,582 |
| BT24 | 45,680,000 | 40,202,768 | 88.01 | 97.34 | 91.69 | 45.7 | 40,192,156 |
| BT25 | 45,760,000 | 40,082,882 | 87.59 | 96.78 | 91.57 | 48.09 | 40,017,094 |
| BT26 | 45,120,000 | 40,234,068 | 89.17 | 96.84 | 91.83 | 46.8 | 37,641,704 |
| BT27 | 44,233,418 | 39,005,056 | 88.18 | 97 | 91.65 | 46.56 | 38,997,352 |
| BT28 | 44,960,000 | 40,140,900 | 89.28 | 96.58 | 90.25 | 45.37 | 39,857,326 |
| BT29 | 45,440,000 | 40,019,690 | 88.07 | 96.79 | 91.49 | 47.93 | 39,928,882 |
| BT30 | 54,720,000 | 40,148,062 | 73.37 | 97.64 | 92.7 | 45.67 | 40,139,212 |

**Note: LS represents 30 fecal samples collected from wild Hainan macaques in Lingshui, and BT represents 30 fecal samples collected from captive Hainan macaques in Baoting.**

**Supplementary Table 2. Pathway Enrichment Scores of Differential Metabolites**

| **Pathway** | **Down** | **Up** | **Metabolite Number** | **DA score** | **State** |
| --- | --- | --- | --- | --- | --- |
| Alanine, aspartate and glutamate metabolism | 0 | 3 | 3 | 1 | Up |
| Alcoholism | 0 | 2 | 2 | 1 | Up |
| Amphetamine addiction | 0 | 2 | 2 | 1 | Up |
| Bile secretion | 3 | 3 | 6 | 0 | Down |
| Cocaine addiction | 0 | 2 | 2 | 1 | Up |
| Dopaminergic synapse | 1 | 2 | 3 | 0.33 | Up |
| Linoleic acid metabolism | 3 | 0 | 3 | -1 | Down |
| Retrograde endocannabinoid signaling | 3 | 0 | 3 | -1 | Down |
| Steroid hormone biosynthesis | 5 | 1 | 6 | -0.667 | Down |
| Tyrosine metabolism | 3 | 2 | 5 | -0.2 | Down |
